# Personalizing cognitive processing therapy with a case formulation approach to intentionally target impairment in psychosocial functioning associated with PTSD

**DOI:** 10.1016/j.conctc.2024.101385

**Published:** 2024-11-01

**Authors:** T.E. Galovski, L.B. McSweeney, R.D.V. Nixon, J.S. Wachen, B.N. Smith, S. Noorbaloochi, D. Vogt, B.L. Niles, S.M. Kehle-Forbes

**Affiliations:** a National Center for PTSD Women's Health Sciences Division at VA Boston Healthcare System, Boston, MA, USA; b Boston University Chobanian & Avedisian School of Medicine, Boston, MA, USA; c Flinders University Institute for Mental Health and Wellbeing, Sturt Rd, Bedford Park, SA, 5042, Australia; d College of Education, Psychology and Social Work, Flinders University, Sturt Rd, Bedford Park, SA, 5042, Australia; e Center for Care Delivery & Outcomes Research, Minneapolis VA Healthcare System, Minneapolis, MN, USA; f Department of Medicine, University of Minnesota, Minneapolis, MN, USA

**Keywords:** Posttraumatic Stress Disorder (PTSD), Cognitive Processing Therapy (CPT), Case formulation, Psychosocial functioning, Veterans, Randomized Controlled Trial (RCT), Evidence-Based Psychotherapy (EBP)

## Abstract

Posttraumatic stress disorder (PTSD) is a debilitating condition often accompanied by significant functional impairments affecting quality of life and well-being. While Cognitive Processing Therapy (CPT) is a leading, evidence-based psychotherapy for PTSD, demonstrating substantial efficacy in core symptom reduction, its impact on psychosocial functioning is less well-established. The Personalizing Cognitive Processing Therapy with a Case Formulation Approach (Personalizing Approaches to Therapy: PATh) study aims to enhance CPT by explicitly targeting functional impairments and idiosyncratic challenges to optimal therapy outcomes (COTOs), comparing its efficacy against standard CPT in improving psychosocial functioning, quality of life, well-being, and core PTSD and depression symptoms. This randomized controlled trial involves 200 Veterans across eight Veterans Health Administration clinical sites, assigned to either Case Formulation CPT (CF-CPT) or standard CPT. Providers will deliver up to 20 sessions per patient, with assessments at baseline, mid-treatment, post-treatment, and three months follow-up. It is hypothesized that Veterans receiving CF-CPT will show greater improvements in functioning, quality of life, well-being, and symptom reduction, alongside higher treatment completion rates compared to standard CPT. Secondary outcomes will examine specific clinical challenges and their influence on treatment outcomes. This study investigates whether a personalized, flexible CPT protocol can enhance functional recovery in PTSD treatment without compromising the efficacy of the traditional approach, potentially impacting clinical practices and patient outcomes by promoting holistic recovery for veterans with PTSD.

## 1 Introduction

Posttraumatic stress disorder (PTSD) is common and complicated and can be chronic and debilitating [1]. The heterogeneity of the twenty core symptoms of PTSD, comorbidity with disorders such as depression, panic, and substance use, high rates of concurrent and lingering effects of physical injury, and suicidality all contribute to complex clinical presentations [[2], [3], [4], [5]]. Additionally, living with PTSD can exact a significant toll on broader domains of well-being such as functioning (e.g. social, occupational, recreational) and quality of life (defined as “individuals' perceptions of their position in life in the context of the culture and value system in which they live and in relation to their goals, expectations, standards, and concerns [6],” p. 1405) even decades after exposure to the trauma [2]. The complex and enduring challenges inherent in PTSD and their effect on patients’ functioning and quality of life pose significant hurdles for patients and clinicians alike [4,7,8].

Cognitive Processing Therapy (CPT) is among the psychotherapies for the treatment of PTSD with the most empirical support to date. ^,^ [[9], [10], [11]] Recent study indicates that CPT has the largest effect size of existing evidence-based psychotherapies (EBPs) for PTSD in military populations [12] and, indeed, across trauma populations (mean ES = 1.69) [13]. Yet the impact of CPT on both core PTSD symptoms and impairments in psychosocial functioning can be improved, as a subset of active duty and Veterans fail to experience clinically meaningful improvement, and only about one-third of patients no longer meet criteria for PTSD posttreatment [11]. In addition, while significant gains in functioning, quality of life, and health–related concerns have been realized in CPT clinical trials with and without Veterans [14,15], these improvements are more modest than those observed in PTSD and depression symptoms [16,17].

Indeed, impairments in psychosocial functioning are important, but less well-attended, facets of PTSD. While significant impairment in functioning is clearly a requirement for the diagnosis of PTSD, as indicated by Criterion G of the Diagnostical Statistical Manual, Edition 5 [18], resolution of functional impairment is not considered to be a *primary* therapeutic target in evidence-based PTSD treatment protocols [19]. Improvements in functioning and, more broadly, quality of life and well-being, are most typically considered secondary outcomes in RCTs, if they are reported at all. This seeming lack of attention to impairments in functioning stands in stark contrast to patients’ reports of the meaningfulness of these impairments in their lives. In fact, it is often precisely these types of impairments that drive patients suffering from PTSD to seek treatment, arguably more so than the 20 core symptoms of the disorder and improvements in these outcomes may be even more clinically meaningful to patients than symptom reduction, as symptom concerns are often raised in the context of their impact on broader functional concerns [[20], [21], [22]]. Given the importance of functional recovery to patients, there is a need to explicitly target these impairments in PTSD treatment protocols.

Despite their importance, functional challenges have historically been difficult to assess and directly target in manualized therapies, perhaps because "functional impairment" is quite variable across patients in breadth and scope [19]. Including *explicit* and manualized instruction on treating every imaginable functional impairment, including the universe of possible clinical challenges to targeting these impairments during the course of treatment for PTSD, is impossible and is likely the reason that no single PTSD psychotherapy exists that is specifically designed to primarily target functional recovery. Indeed, the relatively modest improvements in functional impairments following PTSD treatment may reinforce beliefs that long-term functional goals are beyond the scope of brief therapies and that the pathway to such changes is via treatment reductions in core symptoms of PTSD [[23], [24], [25]].

Suboptimal outcomes and premature dropout from EBPs for PTSD may result, in part, from the lack of flexibility to these types of challenges to optimal therapy outcomes (COTOs) in EBPs [19,26,27]. Importantly, prior research testing other enhancements and expansions to the CPT protocol has resulted in better overall response rates [28] translated to greater engagement in care for a wider variety of patient populations suffering from complicated clinical presentations [[2], [3], [4], [5]], and has *not* compromised the efficacy of the traditional CPT protocol. Specific examples of enhancements to the original 12-session CPT protocol [19,27,28] include RCTs testing a variable length administration of the protocol in which participants are considered completers when they meet good end state functioning (as opposed to a predetermined protocol of 12 sessions) and the possibility of inserting emergency sessions, CPT conducted in one week (massed CPT; Galovski), and delivery of the intervention via telehealth. Examples of enhancements to the protocol include augmenting the protocol with additional interventions to directly target comorbid conditions such as sleep impairment or depression.The accumulation of this research suggests that increasing the flexibility of the CPT protocol to better meet patients’ needs does, in fact, move the needle further toward holistic recovery and does not dilute the efficacy of the traditional protocol.

**Fig. 1:**
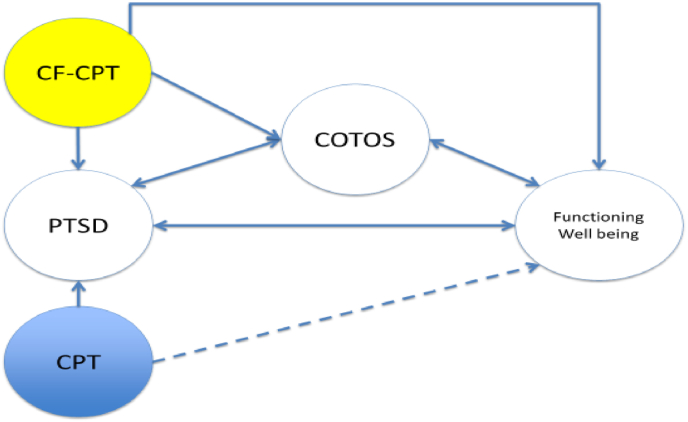
Case Formulation + Cognitive Processing Therapy Conceptual Model *Note:* CF-CPT = case formulation + cognitive process therapy, PTSD = posttraumatic stress disorder; CPT = cognitive process therapy, COTOS = challenges to optimal therapy outcomes.

Case formulation is an approach to treatment that is theoretically consistent with CPT and intentionally seeks to increase the patient's active involvement and agency in his or her own care [29]. The addition of explicit case formulation to the CPT protocol provides a framework for *both* generalizing the effects of CPT to the significant PTSD-related challenges patients face across important domains of functioning *and* intentionally assessing and addressing COTOs as they arise during CPT. In a recently completed open trial designed to assess the efficacy of CF-CPT, (n = 23), results indicated substantial reductions of PTSD, depression, and trauma-related cognitions (*d*s between 1.10 and 1.92 respectively) with excellent maintenance of gains at the three-month follow-up [30]. This treatment approach seeks to increase the effectiveness of CPT beyond PTSD symptoms by expanding the protocol to personalize the approach to clinical care, directly addressing *both* the psychosocial impairments *and* the universe of idiosyncratic challenges that can prevent patients from realizing the full benefits of the intervention and achieving greater overall well-being (see Fig. 1.)

## 2 Research aims and hypothesis

The Personalizing Cognitive Processing Therapy with a Case Formulation Approach to Intentionally Target Impairment in Psychosocial Functioning Associated with PTSD (PATh) study builds on the success of CPT by directly targeting functional impairments and other challenges to optimal therapy outcomes and enhancing overall well-being. Our first aim is to compare the relative effectiveness of case-formulation enhanced CPT (CF-CPT) to CPT in improving psychosocial functioning, quality of life, and well-being as well as core PTSD and depression symptoms. We hypothesize that veterans who receive CF-CPT will demonstrate greater improvements in functioning, quality of life, and well-being as well as greater reductions in PTSD and depression throughout treatment and 3-month follow-up than veterans who receive CPT. Our second aim is to determine the effectiveness of CF-CPT as compared to CPT in improving treatment engagement. We hypothesize that veterans randomized to CF-CPT will demonstrate higher rates of treatment completion than CPT. Lastly, a third aim will evaluate CF-CPT's indirect impact on psychosocial functioning, quality of life, well-being, and PTSD/depression through improvement in the idiosyncratic clinical challenges (COTOs) targeted by the CF. Additionally, we will examine between-group differences in secondary outcomes (e.g., anger, anxiety, health concerns, sleep, numbing/reactivity) and describe the frequency, type, and duration of divergences made by providers. See Fig. 1.

## 3 Materials and methods

### 3.1 Overview

PATh is a two-arm, randomized controlled trial in which VA providers administer CPT or CF-CPT to 200 veterans across 8 VHA medical centers (see Table 2). Each study provider will deliver either CPT or CF-CPT as assigned to up to 15 Veterans presenting for treatment in clinic. Centralized recruitment and enrollment occur at the two coordinating sites, and a local site investigator (LSI) is present at each of the 6 clinical sites. All patients are assessed prior to treatment, mid-treatment, two weeks following the conclusion of treatment, and three months after the post-treatment assessment. Data sources include pre-and posttreatment diagnostic interviews, veteran self-report surveys, administrative data extracted from mental health progress notes, and therapy process data, including daily symptom monitoring diaries and therapy materials. symptoms. Data sources also include standardized measures of documenting clinical challenges that disrupt therapy and are specifically targeted by the CF approach, as well as novel, idiosyncratic measures (daily diaries, therapy materials) of challenges.

**Table 1:** Assessment schedule.

Construct/Domain of Interest	Measure	Interval Assessed	Source
**Aim 1 Outcomes**			
PTSD-related psychosocial functioning (primary)	Inventory of Psychosocial Functioning (IPF) [34]	Pre, Mid, Post, FU	Telephone or VA Video Connect (VVC)
Functioning & disability	World Health Organization – Disability Assessment Schedule 2.0 (WHO-DASII) [36]	Pre, Mid, Post, FU	Online or Mailed Survey
Quality of life	World Health Organization - Quality of Life, Brief (WHOQOL-BREF) [37]	Pre, Mid, Post, FU	Online or Mailed Survey
Well-being	Well-Being Inventory (WBI) [38]	Pre, Mid, Post, FU	Online or Mailed Survey
Clinician-assessed PTSD (primary)	Clinician Administered PTSD Scale-5 (CAPS-5) [31]	Pre, Mid, Post, FU	Telephone or VA Video Connect (VVC)
Self-reported PTSD	PTSD Checklist –DSM-5 (PCL) [39]	Pre, Mid, Post, FU Weekly	Online or Mailed Survey & Therapy Session
Self-reported depression	Patient Health Questionnaire-9 (PHQ-9) [40]	Pre, Mid, Post, FU Weekly	Online or Mailed Survey & Therapy Session
**Aim 2 Outcome**			
Treatment completion	Final session progress note (indicated by use of final session template or note text)	Post	EMR
Treatment Engagement	Therapist Rating Scale	Weekly	EMR
**Potential COTOs/Exploratory Outcomes**			
Comorbid mental health conditions	DSM-5 Level 1 Cross-Cutting Symptom Measure [41]	Pre, Mid, Post, FU	Online or Mailed Survey
Minor life events/hassles	Weekly Stress Inventory [42]	Pre, Mid, Post, FU	Online or Mailed Survey
Health concerns, conditions	Veterans Rand Short Form [43]	Pre, Mid, Post, FU	Online or Mailed Survey
Sleep	Pittsburgh Sleep Quality Index (PSQI) [44]	Pre, Mid, Post, FU	Online or Mailed Survey
Chronic pain	PEG Measure [45]	Pre, Mid, Post, FU	Online or Mailed Survey
Anxiety	State-Trait Anxiety Inventory [46]	Pre, Mid, Post, FU	Online or Mailed Survey
Numbing/reactivity	Emotional Reactivity and Numbing Scale [47]	Pre, Mid, Post, FU	Online or Mailed Survey
Anger	Dimensions of Anger Reactions [48]	Pre, Mid, Post, FU	Online or Mailed Survey
Suicidal Ideation	Beck Suicidal Ideation Scale [49]	Pre, Mid, Post, FU	Online or Mailed Survey
Alcohol & drug use/Craving	Brief Addiction Monitor (use & craving subscales) [50]	Pre, Mid, Post, FU	Online or Mailed Survey
PTSD-related readiness to change	University of Rhode Island Change Assessment [51]	Pre, Mid, Post, FU	Online or Mailed Survey
**Idiosyncratic COTOs (for use in CF-CPT & Aim 3)**			
Idiosyncratic COTOs (CF-CPT only)	Daily Monitoring Diary	Weekly	Therapy Session
**Descriptive Measures**			
Demographics	PhenX Demographic Questionnaire	Pre	Online or Mailed Survey
Trauma History	Locally constructed trauma interview	Pre, Mid, Post, FU	Interview via telephone or VA Video Connect (VVC)

**Table 2:** Veterans Health Administration (VHA) clinical sites.

	Veterans Health Administration (VHA) Clinical Sites
1.	Boston VA Health Care System, Boston, MA^a^
2.	VA Minneapolis Health Care System, Minneapolis, MN^a^
3.	New Orleans VA Medical Center, New Orleans, LA^b^
4.	VA Pacific Islands Health Care System, Honolulu, HI^b^
5.	VA Phoenix VA Health Care, Phoenix, AZ^b^
6.	St Louis VA Medical Center, St Louis, MO^b^
7.	Salem VA Medical Center, Salem, VA^b^
8.	Michael E. DeBakey VA Medical Center, Houston, TX^b^

Note.

a Lead site.

b Local Site.

### 3.2 Participants, inclusion, and exclusion criteria

Participants are 200 male and female veterans seeking routine clinical care in VA. A full diagnosis of DSM–5^18^ of PTSD per the Clinician-Administered PTSD Scale (CAPS-5) [31] is required for inclusion in the study. Study exclusion criteria include suicide or homicide risk that needs to be the immediate focus of treatment, current mania, illiteracy, psychosis, or serious drug or alcohol abuse that requires immediate medical attention (e.g., inpatient care) as determined by chart review and with follow-up questions and discussions with the treating clinician. Patients should not be participating in another trauma-focused therapy at the time of enrollment but can continue any psychiatric medications (dose must be stable for one month prior to enrollment). Medication changes or the addition of other psychotherapies will not be prohibited for veterans in either treatment arm following baseline assessment and randomization. Changes in psychiatric medications and psychosocial treatments during the study period are extracted from the electronic medical record (EMR) at study completion.

### 3.3 Provider randomization, training, and consultation

Providers are VHA clinicians trained in CPT, are listed on the VA CPT National Provider Roster, or are eligible for the CPT roster. Clinicians currently "in training" to become CPT providers are excluded from participation. Up to 45 providers are randomized to deliver either CPT or CF-CPT. Providers only deliver one condition to minimize therapist drift and possible contamination effects.

All providers begin participation in the study with a 3-h training delivered in an interactive online format in groups of up to 8 providers. This training will differ by condition. CPT providers receive a 30-min overview of study-specific procedures (recruitment, tracking, template use) and a refresher of the traditional CPT protocol currently used by the VHA CPT Dissemination Initiative. The CF-CPT group will receive a similar overview of study procedures and explicit training on using the CF approach. The CF-CPT group then receives a second 3-h training delivered one week later, which consists of case examples, role plays, and interactive exercises designed to further instruct the CF-CPT therapists in administering case formulation. To ensure that all therapists maintain fidelity to their assigned treatment, they will attend ongoing weekly consultation led by expert CPT consultants with up to 8 other providers randomized to the same condition. CPT expert consultants are trained in the case formulation approach and have meetings and communication with the PI to ensure adequate guidance in the case formulation model of care.

### 3.4 Veteran recruitment screening, consent, and treatment assignment

To avoid selection bias in the patient population, all consecutive veterans with whom a study provider intends to begin a course of individually delivered CPT are offered study participation. Thus, to be invited to participate, veterans will have been identified as PTSD-positive and eligible for CPT per clinic screening processes. To prevent undue influence and coercion of the patient by the study provider, study staff instruct providers to assure the veterans that participation is voluntary and that there are no consequences to declining participation in the study. Veterans who express interest in the study are contacted by study staff for initial screening to determine potential eligibility, consented, and scheduled for a baseline interview to establish study eligibility. Randomization will occur at the provider, rather than patient, level. Veterans will receive the treatment delivered by their assigned therapist and are informed of their treatment condition prior to consent.

### 3.5 Interventions

Both interventions are delivered weekly. Variable length CPT is now standard practice [9,27,28], and providers in both arms will conclude treatment as indicated by patient progress rather than a prescribed number of sessions (not to exceed 20 sessions or 26 weeks). Although prior research has used 18 sessions (50 % more therapy than the original 12 session protocol allowed) as the maximum number of CPT sessions, 20 sessions was chosen for this trial to allow as much latitude to therapists and patients as possible in completing CPT given the overarching goal of studying the impact of adding sessions to increase the therapists’ ability to address functional impairments and COTOs within the protocol.

#### 3.5.1 CPT

CPT is a cognitive therapy that focuses on challenging and modifying maladaptive beliefs related to trauma. Sessions and daily out-of-session homework consists of helping patients identify and challenge trauma-related beliefs, and later in therapy, overgeneralized beliefs about oneself and the world [9]. Providers delivering CPT are asked to continue to deliver the treatment as they have been in regular clinical practice. The only difference is the additional information requested in the session progress notes which tracks the provider's perception of the Veteran's engagement in the treatment session.

#### 3.5.2 CF-CPT

Traditional CPT explicitly targets the core symptoms of PTSD through the process of cognitive restructuring of trauma-related assimilated and over-accommodated cognitions (or stuck points), targeting impairment in functioning only indirectly through improvement in core symptoms of PTSD. Impairments in functioning are not routinely assessed or incorporated into therapy unless they are trauma-related, and then only at the end of therapy. CF-CPT expands on this protocol to intentionally address impairment in functioning by *directly and intentionally* targeting relevant cognitions throughout therapy, increasing the focus on functional outcomes in a primary fashion. It also enhances the providers' latitude to assess and explicitly address idiosyncratic COTOS that threaten patient engagement in and response to PTSD treatment enables more veterans to realize the full benefits of CPT.

CF-CPT differs from CPT in three ways. First, CF-CPT begins with the case formulation assessment. The case formulation assessment is incorporated into the traditional CPT session 1 (often expanded into two sessions), allowing time to develop the individualized monitoring tool (daily diary). Using a single document titled the “Roadmap to Case Formulation”, the therapist and patient collaboratively first identify strengths and resources that are currently accessible to the patient (e.g. supportive partner or friend, sense of humor). Next, the patient's personal challenges across broad categories are identified. Categories of challenges include 1.) Avoidance (e.g. refusing to talk about the trauma), 2.) Engagement (e.g. many missed sessions in previous courses of therapy), 3.) Emotion regulation (e.g. angry or violent outbursts or suicidality), 4.) Comorbid Mental or Physical Health Concerns (e.g. substance use or traumatic brain injury), 5.) major life stressors (e.g. ongoing child custody dispute or divorce, care of ill family member).

The second way that CF-CPT differs from traditional CPT is in the monitoring of identified COTOs throughout therapy via the daily diary. Patients choose the COTOs from their Roadmap that are most likely to disrupt progress in therapy and add them to a locally constructed daily diary. Patients monitor these COTOs (e.g. experiences of anger) on diaries and rate them on a likert scale from 0 (did not happen today/was not upsetting) to 5 (debilitating or unable to function). The therapist and patient are able to quickly review diaries at the beginning of each session and use that information to identify any elevations in COTOs that might become a treatment priority.

Finally, CF-CPT differs from CPT in the Patients in CF-CPT are instructed to apply CPT-specific skills to COTOs and other functioning-related cognitions throughout practice assignments and treatment sessions (while still prioritizing assimilated stuck points). In addition to these standardized modifications of CPT with case formulation, for some patients in the CF-CPT group, identified COTOs will increase during the course of CPT to a level that threatens progress in the CPT protocol. For only those patients for whom it is necessary (as operationalized in the PATh manual), the content of the therapy may be altered accordingly (including pausing CPT and diverging to another intervention for a specified period of time and then returning to CPT). For example, increases in conditions or symptoms (e.g. suicidality, substance use, anger, sleep impairment) during the administration of the CPT protocol that require immediate and specific (non-trauma-focused) intervention can be prioritized. Together, using the case formulation approach, the patient and therapist can agree to pause CPT and diverge to a different intervention (e.g., hospitalization, affect regulation skill-building, anger management, etc.) to stabilize the COTO for a specified period of time before returning to CPT and completing the trauma-focused work. A more detailed description of the approach is available in Galovski et al. [27].

### 3.6 Treatment fidelity

Adherence and competence are determined by independent and expert raters who are not otherwise involved in the project. This study uses a hybrid expanded CPT fidelity manual (described in Farmer et al. [32]) which more closely tracks the inclusion of CPT elements as well as proscribed elements. The same fidelity manual is used in both conditions to evaluate the presence and/or absence of CF elements. We will randomly select 15 % of treatment session tapes for fidelity rating and determination of inter-rater reliabilities.

### 3.7 Assessment

Participants are assessed prior to treatment (pretreatment), approximately mid-treatment (scheduled to follow session 6 of traditional CPT in order to standardize the timing of this assessment), two weeks posttreatment or 14 weeks after study initiation for treatment dropouts, and 12 weeks after the posttreatment assessment (follow-up). The mid-treatment session timing is approximate given that total treatment length varies across participants due the variable length of treatment format, the potential addition of emergency sessions, and the potential addition of case formulation sessions. Throughout the study, patient data are collected by a) interviews conducted by an independent evaluator blinded to study condition delivered via phone or VA Video Connect (VVC), b) online survey via Qualtrics or, if preferred by veteran, via mailed paper and pencil survey, c) retrieval of data from electronic medical records (EMR) and, d) local site therapy materials collected by clinical site study staff. Table 1 lists the study measure, interval assessed, and domain of interest.

In addition to outcomes specified in the study-specific aims, we will measure constructs and conditions associated with each COTO domain. While we cannot formally assess the universe of possible COTOs for each participant, we selected standardized measures for those most likely to arise. For those randomized to CF-CPT, we will utilize the daily diary completed throughout the course of treatment as an idiosyncratic measure of all relevant COTOs for each individual patient. These could include factors such as interpersonal conflicts, work-related stress, or other functional impairments that may interfere with therapy. These measures provide an overview of baseline severity in these domains and change over the course of the intervention (outcomes that could also be considered COTOs [e.g., depression or functioning] are listed only as outcomes in Table 1).

### 3.8 Analytic plan

Analyses will follow ITT methodology. We will test Aim 1 hypotheses using generalized linear mixed models. Continuous patient outcome measures (Aim 1A & 1B but not dichotomous outcomes of treatment completion), within the assumed and appropriate fitted distributions, are modeled with treatment and training wave as fixed effects and a random provider effect (clustering by provider). The time (pretreatment, mid-treatment posttreatment, three-month follow-up) by treatment interaction term will provide the test of our primary hypothesis that CF-CPT are superior compared to CPT.

For the dichotomous measure of treatment completion (Aim 2), logistic mixed models with a random provider effect are used. Given that the amount of divergence from the CPT protocol will vary across patients, we will augment our primary analysis with secondary analyses comparing subsamples of the CF-CPT group (e.g., those with and without divergences) to the CPT group. This secondary analysis will require adjustments for possible covariate imbalance, as we will lose the balance induced by randomization. We will use propensity analysis and will estimate average treatment effect on the treated (ATT) and the corresponding sensitivity analysis to gain more insight into the magnitude of the effect. Post hoc power will also be calculated to assess the generalizability of these estimates.

Finally, we will examine the COTO reduction score as a predictor of improvements in functioning and symptoms – i.e., testing whether more improvement in COTO scores is associated with greater improvement in study outcomes (Aim 3). Using the daily diaries, we will calculate a single index, called a Composite Primary COTO Reduction (CPCR) score, following the method of Blanchard & Schwarz [33]. This score will serve as an index of overall change in COTO level and serves the function of reducing potential Type I error from analyzing multiple COTOs singly (allowing patients and providers to track as many COTOs as necessary). It can be conceptualized as a percentage of improvement. The following formula is used to calculate the CPCR score. The symptoms used in the calculations are examples only.

$$COTO\;Reduction\;Score\;\left(CRS\right):\frac{Average\;pretreatment\;anger\;outbursts\;ratings-average\;anger\;outbursts\;ratings}{Average\;pretreatment\;anger\;outburst\;ratings}$$

A CRS is calculated for each COTO endorsed. CRS scores are then used to calculate overall CPCR scores:

**Anger reduction score + urge to fight reduction score + etc.**

**CPCR score = 6 (or 7 or 8, etc.) (depending on number of COTOs present)**

These CPCR scores can be calculated on the entire diary or any subsection. For instance, a CPCR score could be calculated for overall improvement or on improvement on subdomains of psychosocial functioning. We will include CPCR (as a measure of improvement in COTOs) as well as scores on relevant standardized assessments and an interaction between these scores and time in the multi-level model we propose.

#### 3.8.1 Power and sample size

With an anticipated sample size of 200 veterans and an estimated standard deviation (SD) of 17.01 for the total IPF score [34] we will have at least 80 % power to detect a difference of 8.49 in mean psychosocial functioning scores for alpha = 0.05 and a 1:1 sample size. With respect to examining symptom improvement, estimating SD = 14.5 and the same design and alpha functions for the analyses listed above, we will have 80 % power to detect a minimum of 7.24-point difference between the group means on the CAPS-5.

## 4 Discussion

Left untreated, PTSD is often chronic and associated with significant functional impairments, medical morbidity, and high levels of healthcare utilization. Fortunately, treatment with evidence-based therapy significantly reduces the suffering and costs associated with PTSD. However, there is a great need to increase flexibility and person-centeredness within the CPT approach. Case formulation is an approach to treatment theoretically consistent with CPT and intentionally seeks to improve the veteran's active involvement and agency in their own care [27,29]. This study will ascertain whether increasing the flexibility of CPT to target COTOs will positively impact veterans' health and well-being, enabling more veterans to fully benefit from CPT and expanding its impact on functional outcomes.

This is the first large-scale clinical trial designed to incorporate case formulation into an EBP for PTSD in an effort to provide guidance for clinicians to 1) assess the universe of possible COTOs for their patients, 2) continuously monitor the status of those COTOs during treatment, and 3) apply this information to inform treatment decisions including continuing with the CPT protocol or diverging to stabilize the COTO and then returning to the protocol. Although multiple trials have been conducted to *enhance* (test modifications to the format of the core protocol) and *expand* (and new elements or augmentations to the core protocol) CPT, this approach tests a *personalized approach* to the protocol. If successful, this case formulation approach to CPT will provide guidance and parameters regarding how to address functional challenges to optimal treatment outcomes as patients navigate recovery from PTSD. In addition to employing methodologically rigorous standards for clinical trials (reliable and valid standardized measures, gold standard diagnostic interviews, blinded assessments, independent rating of fidelity by experts, long-term follow assessment, and conducting intent to treat analyses), we are also assessing and measuring the universe of possible COTOs through the use of personalized daily diaries. Providers will also complete independent ratings of each weekly COTO. Calculating composite scores form these diaries will allow us to understand the impact of the COTOs on the course of CPT as well as to evaluate the effectiveness of the intervention on the COTO. The engagement of multiple sites across the VA healthcare system and the naturalistic recruitment and engagement of study participants is another strength of the study. The broad inclusion criteria and the use of field-based providers in these eight VA outpatient clinics will increase the generalizability of the results. This multifaceted approach allows for a detailed understanding of the impact of CF-CPT on veteran psychosocial functioning,

This trial is not without limitations. The unique nature of the design, tracking idiosyncratic COTOs for participants in the CF-CPT condition to inform treatment decisions, provides excellent, nuanced data on the experimental condition. However, utilizing the same tracking system in the control condition is not possible as it would alter the treatment as usual. This precludes direct comparison of the participants’ COTOs and improvements on those COTOs across conditions. Therapist assessment of and attention to COTOs in the control condition may be obscured simply due to lack of measurement. To compensate, for all participants at each assessment interval, we include standardized instruments assessing the most common COTOs and an expanded, locally constructed interview probing likely COTOs and functional impairments in depth to detect presence and change over time. We have created a template querying divergences from session content and capturing functional impairments for therapists randomized to each condition to complete at the conclusion of each session. Finally, study staff are present at all therapist consultation calls to capture additional data about COTOs, functional impairments, any divergences from protocol, reasons for premature drop-out, etc. Premature drop-out is defined as ending therapy prior to session 12 of traditional CPT except in cases of early response to treatment (e.g. loss of PTSD diagnosis, scores below a 25 on the PCL-5, and agreement on ending treatment by therapist and patient) [35]. Finally, while multi-site design enhances generalizability, all therapists are field-based and have been trained within the VA and conduct CPT as usual within the parameters set by the VA national CPT training initiative. As a result, these results may not translate well outside the VA and to non-veteran populations.

This randomized control trial represents a significant advancement in PTSD treatment by integrating a case formulation approach to CPT. If successful, the PATh model of care will allow for more flexible administrations of EBPs for PTSD without compromising their efficacy. By intentionally focusing on functional impairments as a primary target of the intervention and flexing the administration of the CPT protocol when the amplification of COTOs warrant additional attention, we hope to 1) reduce premature attrition thereby ensuring an adequate dose of therapy, 2) reduce impairment in identified functional impairments, and 3) move the needle further toward holistic recovery.

## Funding

This work was supported by the Department of Veteran Affairs (award number RX003369-01A1).

## CRediT authorship contribution statement

**T.E. Galovski:** Writing – review & editing, Writing – original draft, Methodology, Investigation, Funding acquisition. **L.B. McSweeney:** Writing – review & editing, Writing – original draft, Project administration. **R.D.V. Nixon:** Writing – review & editing, Writing – original draft, Methodology, Conceptualization. **J.S. Wachen:** Writing – review & editing, Writing – original draft, Investigation. **B.N. Smith:** Writing – review & editing, Writing – original draft, Methodology, Investigation. **S. Noorbaloochi:** Writing – review & editing, Writing – original draft, Methodology, Investigation. **D. Vogt:** Writing – review & editing, Writing – original draft, Methodology, Investigation. **B.L. Niles:** Writing – review & editing, Writing – original draft, Methodology, Investigation. **S.M. Kehle-Forbes:** Writing – review & editing, Writing – original draft, Methodology, Investigation, Funding acquisition.

## Declaration of competing interest

The authors declare that they have no known competing financial interests or personal relationships that could have appeared to influence the work reported in this paper.
